# Validity and reliability of serratus anterior hand held dynamometry

**DOI:** 10.1186/s12891-019-2741-7

**Published:** 2019-08-07

**Authors:** Jos IJspeert, Hans C. J. W. Kerstens, Renske M. J. Janssen, Alexander C. H. Geurts, Nens van Alfen, Jan T. Groothuis

**Affiliations:** 1 Department of Rehabilitation, Donders Institute for Brain, Cognition and Behaviour, Radboud University Medical Center, Nijmegen, the Netherlands; 2 Department of paramedical studies, HAN University of Applied Sciences, Nijmegen, The Netherlands; 3 Department of Neurology and Clinical Neurophysiology, Donders Institute for Brain, Cognition and Behaviour, Radboud University Medical Center, Nijmegen, The Netherlands; 4 IQ Healthcare, Radboud Institute for Health Sciences, Radboud University Medical Center, Nijmegen, The Netherlands

**Keywords:** Muscle strength dynamometer, Strength testing, Reproducibility of results, Serratus anterior, Scapula

## Abstract

**Background:**

Strength testing of the serratus anterior muscle with hand held dynamometry (HDD) in supine subjects has low reproducibility, and is influenced by compensatory activity of other muscles like the pectoralis major and upper trapezius. Previously, two manual maximum voluntary isometric contraction tests of the serratus anterior muscle were reported that recruited optimal surface electromyography (sEMG) activity in a sitting position. We adapted three manual muscle tests to make them suitable for HHD and investigated their validity and reliability.

**Methods:**

Twenty-one healthy adults were examined by two assessors in one supine and two seated positions. Each test was repeated twice. Construct validity was determined by evaluating force production (assessed with HHD) in relation to sEMG of the serratus anterior, upper trapezius and pectoralis major muscles, comparing the three test positions. Intra- and interrater reliability were determined by calculating intra-class correlation coefficients (ICC) smallest detectable change (SDC) and standard error of measurement (SEM).

**Results:**

Serratus anterior muscle sEMG activity was most isolated in a seated position with the humerus in 90° anteflexion in the scapular plane. This resulted in the lowest measured force levels in this position with a mean force of 296 N (SEM 15.8 N). Intrarater reliability yielded an ICC of 0.658 (95% CI 0.325; 0.846) and an interrater reliability of 0.277 (95% CI -0.089;0.605). SDC was 127 Newton, SEM 45.8 Newton.

**Conclusion:**

The results indicate that validity for strength testing of the serratus anterior muscle is optimal with subjects in a seated position and the shoulder flexed at 90° in the scapular plane. Intrarater reliability is moderate and interrater reliability of this procedure is poor. However the high SDC values make it difficult to use the measurement in repeated measurements.

## Background

The ability to stabilize the scapula against the chest wall at rest and during upper limb movement has been widely recognized as a prerequisite for optimal upper limb function and related daily activities [1, 2]. Scapular dyskinesis, defined as abnormal scapular position and movement that may result in e.g. ‘winging’ or ‘tipping’, has been observed in many types of shoulder pathology, such as impingement syndrome, rotator cuff and labral tears, glenohumeral instability, and secondary to central and peripheral nervous system disorders [3–6]. Several authors have related scapular dyskinesis to loss of muscle strength in the scapulothoracic muscles, such as the lower and middle parts of the serratus anterior muscle [7–9]. Lack of strength or endurance in this muscle can cause downward (medial) rotation of the scapula, making its lower medial border more prominent [10]. Others have related scapular dyskinesis to a muscular imbalance (or discoordination) rather than muscle weakness [2, 6, 11]. Yet, various rehabilitation programs promote scapular strengthening exercises in the treatment of patients with shoulder disorders [12, 13]. However, reference values for serratus anterior muscle strength are not available. In addition, a strength training approach may not be beneficial for patients who have coordinative problems [14–16]. In this perspective, it is important to test serratus anterior muscle strength and coordination separately in order to differentiate between patients who can and those who cannot benefit from strength training. The presence of scapular dyskinesis in the absence of strength loss would suggest that motor control therapy might be a more successful approach than strength training.

Manual muscle strength is routinely scored using a six point scale described by the Medical Research Council (MRC) [17]. Although the use of the MRC scale is widespread, its usefulness and reliability is questionably, particularly around joints other than the elbow and knee [18, 19]. Especially the evaluation of relatively normal muscle strength within the upper ranges of the MRC scale lacks interrater reproducibility [18], which is understandable as the definitions of the MRC grades imply that grade 3 is a fixed point (‘anti-gravity strength’), but grade 4 is a wide range between grade 3 and ‘normal’ muscle strength (grade 5) [17]. Handheld dynamometry (HHD) was demonstrated to be a reliable alternative for MRC testing of muscle strength [20–22]. Reliability of HHD has been found to be high for the serratus anterior and trapezius muscles, although its validity with regard to these muscles has not been extensively studied [23].

Serratus anterior muscle strength is commonly evaluated by applying axial pressure to the humerus in the frontal plane with subjects in a supine position and their scapula protracted with 90° of anteflexion in the shoulder [24]. Due to the protraction, the supine test position might be prone to recruiting muscle activity in the pectoralis muscles and therefore not suited for measuring isolated serratus anterior muscle strength [24, 25]. Evaluation of serratus strength using active horizontal abduction during testing to correct for pectoralis activation is possible [26], however not feasible in clinical practice. Ekstrom et al. presented a different approach to the evaluation of maximum voluntary isometric contraction (MVIC) of the serratus anterior muscle. Their subjects were sitting upright with lumbar support, the arm positioned in the scapular plane and in 90° or 125° anteflexion of the shoulder [27]. In these two positions resistance was applied in the scapular plane at the olecranon and at the inferior angle of the scapula attempting to rotate the scapula downward (medially) [27]. They reported significantly higher surface electromyography (sEMG) activity of the serratus anterior muscle during MVIC testing in both seated positions compared to the supine position [27]. However, the two seated testing positions presented might also lack validity. The force needed for the serratus test above 90° in the scapular plane can produce co-contraction of the trapezius descendens muscle [25, 28]. Therefore, the strength found in these positions is most likely not produced by the serratus anterior muscle alone.

To allow valid and reliable strength testing of the serratus anterior muscle with HHD, we modified both seated test positions described by Ekstrom et al. [27]. This modification was needed because the original tests required two points of contact, whereas HHD is only possible with one point of contact. Because of the lack of reference values it was not possible to compute a reasonable force production expected for these test positions. Therefore, construct validity was determined in healthy adult subjects, during MVIC testing, by evaluating sEMG activity of the serratus anterior, upper trapezius and pectoralis major muscles for the two modified seated positions compared to the supine position. We hypothesize that the position with the most isolated serratus anterior muscle EMG activity constitutes the most valid test for serratus anterior strength. In addition, for force measurements with HHD, intra- and interrater reliability of each test position were evaluated by comparing repeated measurements by two assessors.

## Methods

This study was approved by the medical ethical committee of the Radboud University Medical Center and complied with the declaration of Helsinki. Written informed consent was obtained from each subject before inclusion in this study.

### Subjects

Twenty-one healthy subjects were recruited by convenience sampling from physical therapy students of the University of Applied Sciences in Nijmegen, the Netherlands.

Inclusion criteria were: age 18 years or older and sufficient knowledge of the Dutch or English language to understand written and spoken instructions. Exclusion criteria were: rotator cuff tendinopathy or tears, other glenohumeral or subacromial deficits, rheumatic diseases, central or peripheral nervous system disorders, acute shoulder pain before or during the experiment. All above criteria related to the tested arm (right side), if applicable.

### Experimental protocol

**Fig. 1:**
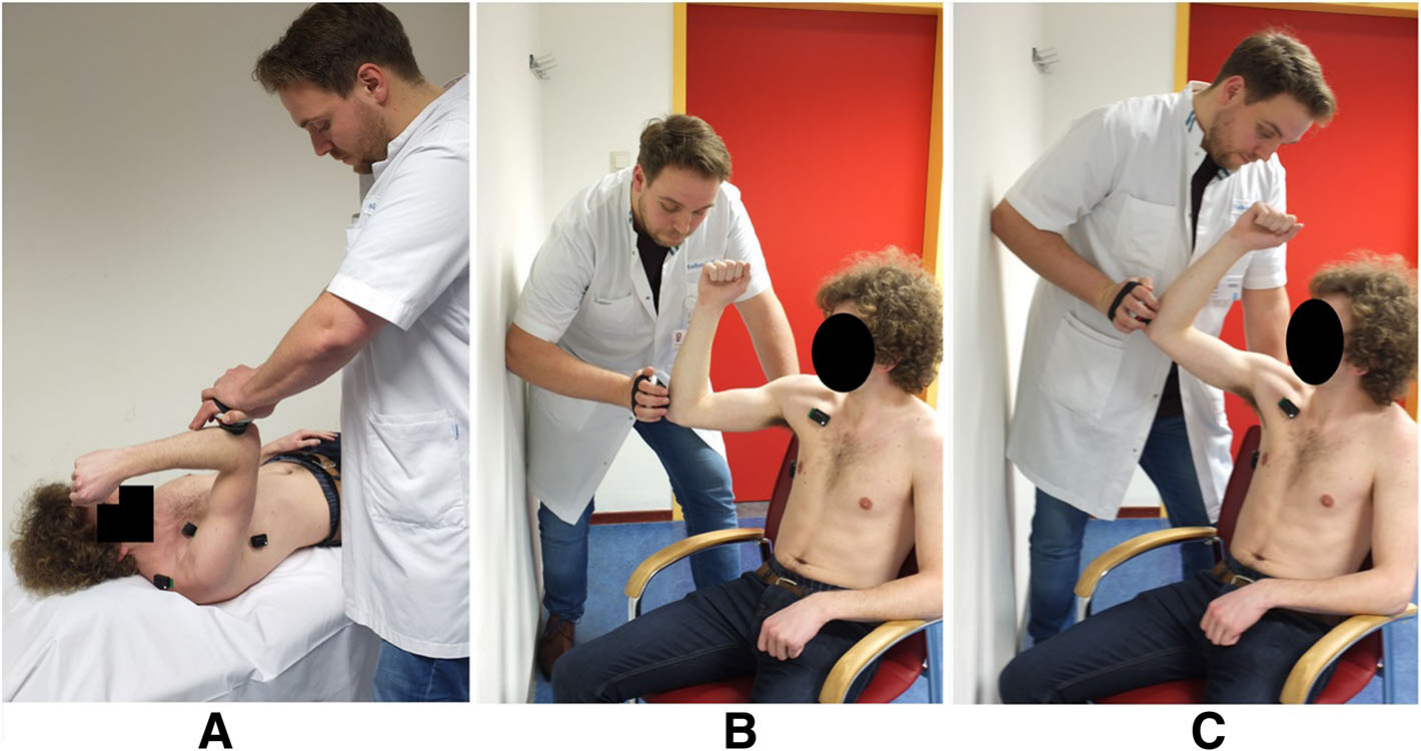
Test positions and surface electromyography placements for serratus anterior muscle testing, note; depicted angles differ from actual goniometry angles measured while testing. **a** 90°of shoulder flexion in the frontal plane. **b** 90° of shoulder flexion in scaption. **c** 125° of shoulder flexion in scaption

We examined three different positions (A, B and C) to test the muscle strength of the serratus anterior. Test position A is a frequently used evaluation of serratus anterior muscle strength described by Michener et al. [24]. In supine position, subjects are required to resist strength applied by the HHD placed just below the olecranon, while placing the elbow and the shoulder in 90° flexion in the frontal plane. Test positions B and C have been derived from Ekstrom et al. and adapted for use with HHD [27]. Subjects are seated in a stable chair with lumbar support, but without scapular support. They are instructed to elevate the tested arm in the scapular plane to respectively 90° and 125° shoulder flexion, with the elbow in 90° of flexion. Angles were checked with a standard goniometer. Axial pressure was applied with the HDD on the olecranon in the scapular plane. In test positions B and C, assessors placed themselves against a wall for extra stability and strength, in contrast to position A. (see Fig. 1).

### Measurements

Subjects were tested during one day in the morning and afternoon. All subjects refrained from any sports activity on the day of testing.

For strength and sEMG measurements the ‘make method’ for strength testing was used [29]. MVIC testing was carried out by two assessors, both experienced physical therapists (JI, 30 yrs.,115 kg,192 cm; and HK, 40 yrs.,76 kg,183 cm). Calibrated Microfet II™ HHDs were used for the collection of strength data. A test assistant stored the sEMG output as well as the strength data in Newton on a computer. The assessors were not able to read out strength data or sEMG signals during testing. Per test position, each contraction lasted 3 s with a ramp up of 4 s and a ramp down of 4 s. Starting cues for timing of the tests were given by the test assistant. The assessors instructed subjects as follows: after the given cue for start of measurement, they counted down from 4 to 0, after which the subject was asked to “push-push-push” for 3 s and then asked to gradually release strength while counting down from 4 to zero. Each contraction was repeated twice per test position, with at least one-minute rest between trials. Every subject was tested twice by each assessor, with a two-minute resting period in between, in test positions A, B and C.

### EMG setup

Surface EMG signals were collected from the serratus anterior, upper trapezius and pectoralis major muscles by applying wireless sensors (W4p-SP-W01, Delsys Inc., Boston, USA) to the skin with Delsys Adhesive Sensor Interface. Prior to electrode placement, the skin was carefully shaved, degreased with alcohol and rubbed with sanding paper. The sEMG sensors were placed at the following locations: for serratus anterior muscle measurement at the 6th to 8th rib in the mid-axillary line anterior to the fibers of the latissimus dorsi muscle [27]; for upper trapezius muscle measurement at 50% on the line from the acromion to the C7 spinous process, following SENIAM guidelines [30]; and for the pectoralis major muscle measurement electrodes were placed approximately 2 cm medial to the coracoid process (Fig. 1, [28, 30]).

### Data analysis

The sEMG signals were filtered and rectified (low cut-off filter 10 Hz, high-cut off filter 1000 Hz, notch filter at 50 Hz), digitized at a sampling rate of 2000 Hz with a common mode rejection ratio of > 80 dB (W4p-SP-W02, Delsys Inc., Boston, USA), and were stored on a laboratory computer for offline analysis. During offline analysis, the root mean square (RMS) of the sEMG signals during the three second maximum for each contraction were calculated using EMG Works**®** (Delsys Inc., Boston, USA). Subsequently, the signals of the 2 contractions per test position were averaged per assessor. Strength data was recorded in Newtons.

### Statistical analysis

#### Validity

Statistical analysis was performed using Statistical Analysis System 9.2 (SAS Institute Inc., Cary, USA). Inspection of sEMG data revealed a non-normal distribution. Therefore, a logarithmic transformation was performed to correct for skewness. A linear mixed model for repeated measurements was used to assess the differences between the three test positions for each muscle, separately. The model reference point was set at test position C, as it was estimated that this test position would produce most serratus anterior muscle activity based on the study by Ekstrom et al. [27]. The dependent variable was the logarithmically transformed RMS-value of EMG activity during the 3-s maximum contraction. The estimated values of the sEMG activity for each position and the relative differences between the positions with 95% confidence intervals (CIs) were calculated by use of the anti-logarithmic transformation.

#### Reliability

Strength data was also inspected for normality. The difference in muscle force (N) between the test and the retest measured by JI, and between tester JI and HK was calculated. Reproducibility (test-retest) was divided into assessment of reliability and agreement parameters [31]. Reliability was analysed using the intraclass correlation coefficient (ICC). ICC’s were calculated using a two-way mixed effect model (ICC3.1agreement) with 95% confidence intervals (CI). ICC values were interpreted as follows in terms of reliability: < 0.5 as “poor”, 0.5–0.75 as “moderate”, 0.75–0.9 as “good”, and > 0.9 as “excellent” [32]. To assess agreement, the standard error of measurement (SEM agreement) and the smallest detectable change (SDC agreement) were calculated. Both were expressed in the unit of the measurement, Newton. The SEM was calculated as SEM agreement = pσ2 error = p(σ2 o + σ2 residual) [33]. The variance due to systematic differences between the observers (σ2 o) and the residual variance (σ2 residual) were obtained through a varcomp analysis [33]. The SEM agreement was used to calculate the SDC agreement = 1.96 pn SEM [30]. In this formula ‘n’ refers to the number of measurements, which is two in our study for test-retest reliability and inter-tester reliability [30]. Bland-Altman plots were constructed to determine if there was bias in measurement error [34, 35]. This plot shows the rater difference against the mean muscle force. The plot visualizes the relationship between the measurement error and the observed value including the presence of systematic bias and bias related to the magnitude of serratus anterior strength. The 95% limits of agreement (95% LoA) were shown in the plot (mean difference ± 1.96 SD). All analyses were performed using IBM SPSS Statistics v22 (SPSS Inc., Chicago, Illinois, United States).

## Results

We included 21 subjects (15 males; 19 right-handed) with a mean age of 21 years (range 19–32) and a mean (± SD) BMI of 22.7 ± 2.1 kg/m^2^. Mean forces (± SEM) measured per test position were: position A 369.8 ± 18.3 N; position B 296.0 ± 15.8 N; and position C 313.0 ± 19.8 N.

**Fig. 2:**
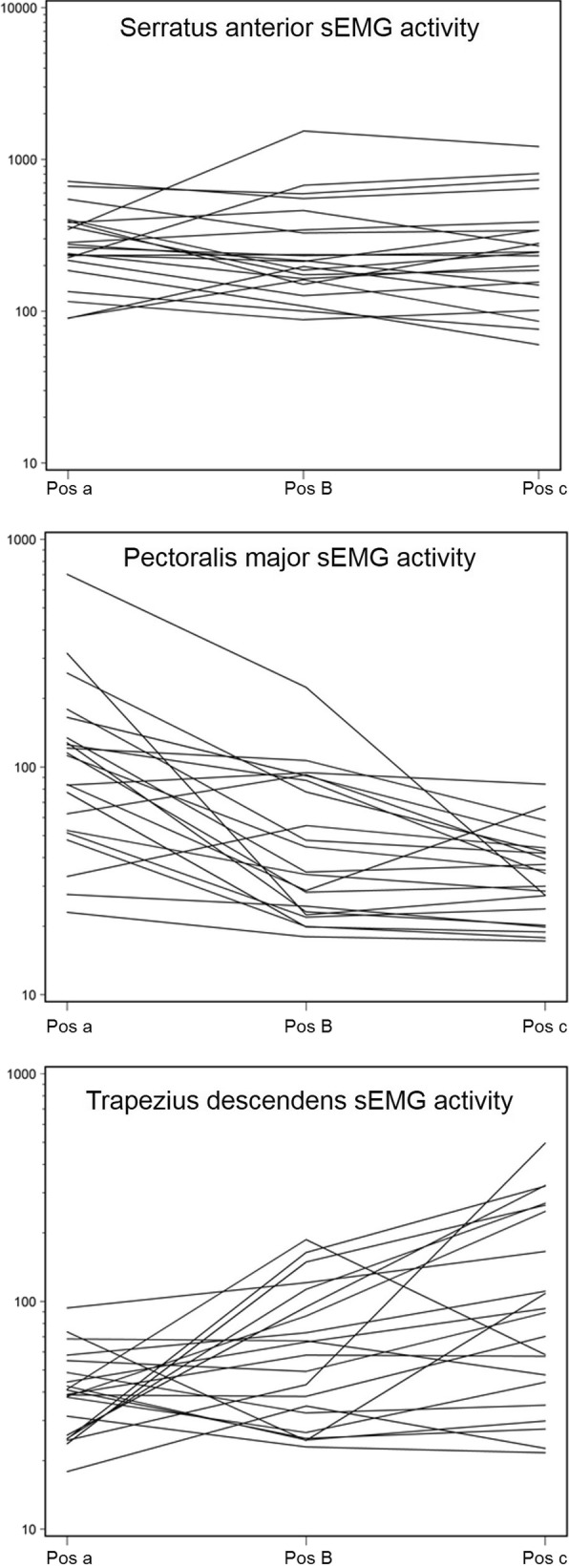
Linear mixed models analysis of EMG activity in micro Volts. EMG, electromyography; Pos A, test position A; Pos B, test position B; Pos C, test position C. Note: the scaling for serratus anterior sEMG activity is different

sEMG activity of the serratus anterior muscle was very similar between the three different test positions. However, the pectoralis major muscle showed significantly more activity in position A compared to B and C, and the upper trapezius muscle showed significantly more activity in position C compared to A and B (see Table 1, Fig. 2).

**Table 1:** linear mixed models of electromyography activity difference estimations

Model	Effect	Estimate	95% CI	
			LL	UL
Serratus anterior	Intercept (mV)	239.54	173.78	330.13
	Pos A	1.09	.78	1.38
	Pos B	1.02	.81	1.24
	Pos C	1	–	–
Pectoralis minor	Intercept (mV)	33.04	23.23	45.04
	Pos A	2.94	2.13	4.05
	Pos B	1.32	.96	1.83
	Pos C	1	–	–
Trapezius descendens	Intercept (mV)	89.88	28.34	53.82
	Pos A	.43	.29	.65
	Pos B	.63	.42	.95
	Pos C	1	–	–

Legend: 95% CI, 95% confidence interval; LL, Lower limit; UL, upper limit; mV, microvolts; Pos A, test position A; Pos B, test position B; Pos C, test position C (reference)

ICC values and agreement parameters for test-retest and interrater reliability of test positions A, B and C are reported in Table 2.

**Table 2:** test-retest and interrater reproducibility of serratus anterior hand held dynamometer strenght testing

	*mean (SD)*	*mean (SD)*	*mean (SD)*		*(95% CI)*	*(N)*	*(N)*
*test-retest*							
Pos A	383.88 (77.65)	404.92 (84.96)	−21.05 (60.22)	− 139.08; 96.98	0.712 (0.420; 0.871	44,10	122,40
Pos B	314.43 (75.1)	322.34 (82.34)	−7.90 (65.90)	− 137.06; 121.26	0.658 (0.324; 0.846)	45,80	127,00
Pos C	351,54 (100.36)	376.16 (93.55)	−24.62 (59.37)	− 140.99; 91.75	0.794 (0.490; 0.916)	44,52	123,40
	*Tester JI*	*Tester HK*	*Diff Tester JI* VS *HK*				
*Interrater*	*mean (SD)*	*mean (SD)*	*mean (SD)*				
Pos A	383.88 (77.65)	340.44 (82.95)	43.43* (92.74)	− 138.34; 225.20	0.794; (0.552; 0.912)	55,80	196,80
*Pos B*	314.43 (75.10)	264.21 (61.76)	50.21* (78.85)	− 104.34; 204.76	0.277 (− 0.089; 0.605)	55,80	154,50
Pos C	351.54 (100.36)	265.10 (40,0))	86.48* (86.21)	−82.49; 255.45	0.226 (−0.107; 559)	85,31	236,47

*N* Newton, *SD* Standard deviation, *Diff* Difference, *LoA* limits of agreement, *ICC* Intraclass correlation coefficient, *CI* Confidence interval, *SEM* Standard error of measurement, *SDC* Smallest detectable change, *%* percentage, * *p* < 0.001

Legend: *ICC 3.1* intraclass correlation coefficient model 3.1, *95% CI*, 95% confidence interval, *LL* Lower limit, *UL* upper limit

**Fig. 3:**
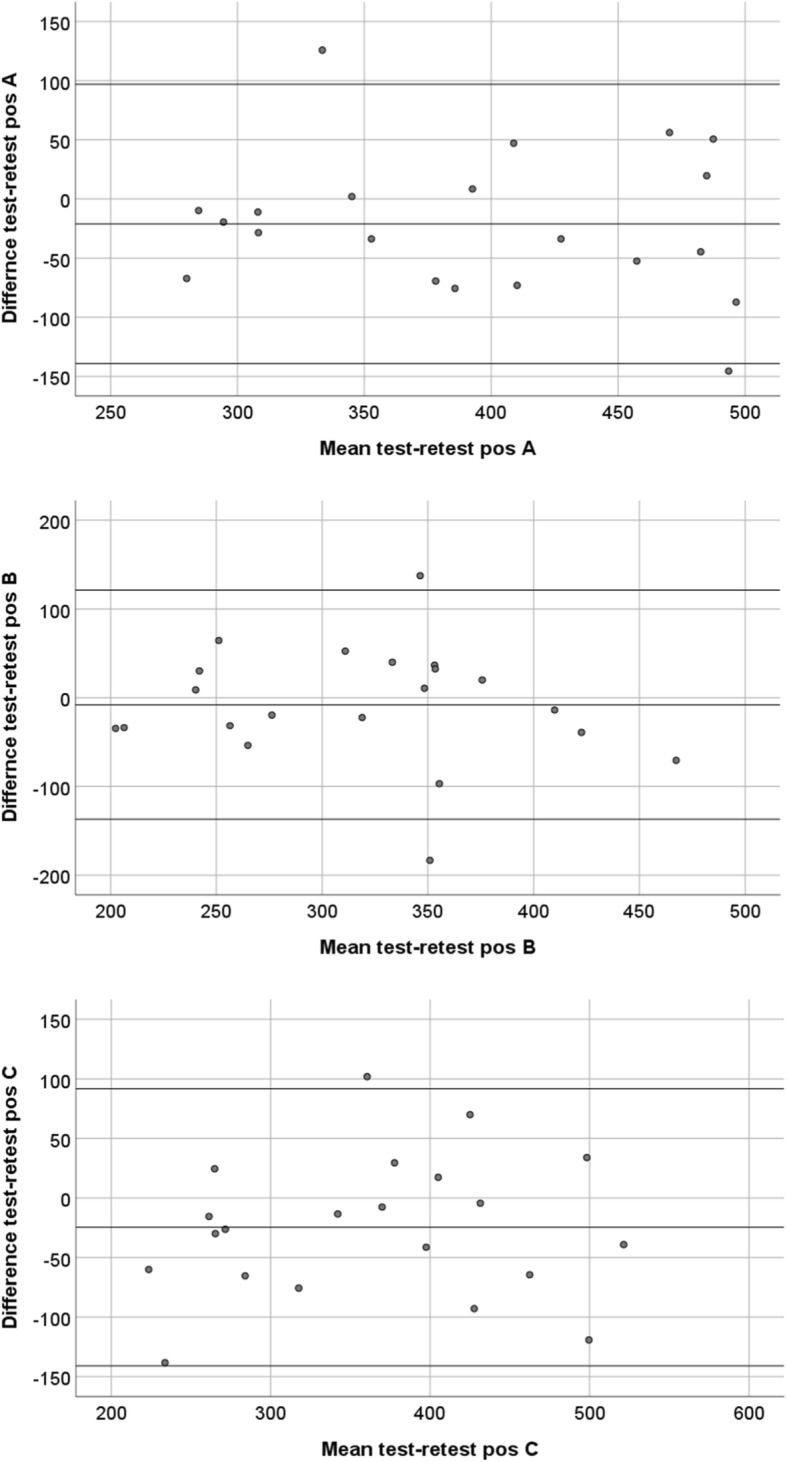
Bland Altman plots for test-retest differences and their relation to the magnitude of strength measured with HHD in Newtons

Paired samples t-tests for the difference scores between HK en JI, were significantly different (*p* > 0.05), showing no agreement between these different raters. Therefore Bland-Altman plots were only presented for the test-retest data (Fig. 3).

## Discussion

By using sEMG of the serratus anterior, upper trapezius and pectoralis major muscles we were able to demonstrate that the serratus anterior muscle was equally activated in three test positions, but most selectively in the seated position with the arm placed in 90° of flexion in the scapular plane (position B, Fig. 1). Position B produced less maximum strength compared to position A (− 74 N) and C (− 17 N), suggesting less co-contraction by the upper trapezius or pectoralis major muscles. Therefore, test position B seems to be the most valid position for isolated assessment of serratus anterior muscle strength measured with HDD. We found similar sEMG activity in all test positions, although we expected to measure most serratus anterior muscle activity in position C based on the previous study by Ekstrom et al. [27]. An important difference with the present study is that the original test provides the opportunity to apply resistance to the arm as well as the scapula using two hands. We used only one point of contact at the arm without scapular fixation, which can explain more similar serratus anterior muscle activity among test positions in our study. The idea of testing in the scapular plane is supported by a recent cadaver study, which has shown that the serratus anterior muscle fascicles from the 4th to 9th rib are attached to the inferior angle of the scapula [36]. The inferior angle of the scapula shows more movement when the arm moves in the scapular plane than in the frontal plane [37]. This confirms that serratus anterior muscle strength should be tested in the scapular plane.

We observed a moderate intrarater reliability (ICC3.1 agreement). Intrarater reliability of test position B was moderate, although somewhat lower than of position A, with an ICC of .658. Interrater reliability was poor with an ICC of .277. However, the SDC agreement and SEM agreement are rather large. The apparent contradiction between a moderate ICC 3.1 agreement and high SDC agreement and SEM agreement is likely to be caused by the high heterogeneity in the population variance, which makes the random error and systematic error relatively lower. However, and SDC agreement of 127 N for the most valid test position (position B) makes it less fit for use in test-retest settings.

The interrater reliability shows low ICC 3.1 agreement. Moreover, the T-test difference in measurements done by HK and JI was significant (*P* > 0.05), so there is no agreement in these measurements (supported by even higher SDC agreement and SEM agreement scores).

The Bland-Altman plots did not show any systematic error in measurement, but did show increased difference scores in the high strength measurements.

We found relatively low intra- and interrater agreement in all our tests. Our subjects produced strength values exceeding 290 N. Although we tried to compensate for this by placing the assessor’s arm holding the HHD against a wall in positions B and C, our approach may still have led to variation between raters resulting in only fair to moderate ICC values. Another factor may have been the different physical characteristics of the two assessors in our study, who had a substantial difference in body size and weight and therefore, possibly, a different ability to provide resistance to the subject’s force production. When compared to the data reported by Michener et al., the ICC values found in the present study are relatively low [24], but the strength values are much higher (exceeding 290 N compared to around 150 N [24]). This may be caused by the fact that Michener et al. included subjects with shoulder pain, whereas we tested healthy young volunteers. It has previously been reported that the reliability of HHD decreases with strength testing levels above 120 N [38]. This may be due to the fact that assessors do not have sufficient strength to resist the force produced by the subject.

Translating our results to patients with shoulder problems, the reliability of the measurements is likely to improve in impaired subjects, because smaller amounts of strength are required from the assessor to counteract the serratus anterior muscle forces. Using a stabilization device, as was done in a study by Kolber et al., might also improve reliability, but will decrease the feasibility of the proposed testing protocol in clinical practice [39].

Our study had some limitations. First, we used sEMG instead of finewire-needle EMG signals, to avoid subject discomfort and for medical-ethical reasons. Although sEMG captures a larger number of motor units compared to finewire needle EMG, the use of surface electrodes might have resulted in cross-talk [40]. For instance, for the upper trapezius muscle, cross-talk might occur from the underlying supraspinatus muscle, and for the serratus anterior muscle from the intercostal muscles. Yet, studies by Fuglevand et al. and Winters et al. indicated that 90% of the sEMG signal is recorded from within 10–12 mm of the surface electrodes when an electrode spacing of 20 to 25 mm is used [40, 41]. In our setup this approach should have provided sufficient confidence to measure relatively isolated sEMG data from the serratus anterior, pectoralis major and trapezius descendens muscles, because the musculature possibly causing cross-talk was located well away from this distance. Another limitation may have been the difference in physical characteristics between the two assessors in this study. However, such variations will also occur in regular clinical practice. Finally, the study group of 21 subjects falls short of the proposed 30 subjects or more by cosmin standards [42]. We feel that the validity part of the study has not suffered from the lower number of subjects.

## Conclusion

We recommend to assess serratus anterior muscle strength manually, applying axial pressure to the humerus, with subjects in a seated position and with the shoulder flexed at 90° in the scapular plane. Given the relatively low agreement parameters, evaluation of treatment with HHD should preferably be done by the same assessor. Although further research validating this test procedure in patients with shoulder complaints and pathologies is needed, we expect it to be more feasible in populations with shoulder problems because of limited strength values in those groups.

## Data Availability

The datasets used and/or analysed during the current study are available from the corresponding author on reasonable request. The dataset has not been posted in an online depository.

## Abbreviations

CI: Confidence interval
HHD: Hand held dynamometry
ICC: Intraclass correlation coefficient
MVIC: maximum voluntary isometric contraction
RMS: Root mean square
SEM: Standard error of the mean
sEMG: Surface electromyography
