# Correction to: Validity and reliability of serratus anterior hand held dynamometry

**DOI:** 10.1186/s12891-019-2780-0

**Published:** 2019-09-17

**Authors:** Jos IJspeert, Hans C. J. W. Kerstens, Renske M. J. Janssen, Alexander C. H. Geurts, Nens van Alfen, Jan T. Groothuis

**Affiliations:** 1 Department of Rehabilitation, Donders Institute for Brain, Cognition and Behaviour, Radboud University Medical Center, Nijmegen, the Netherlands; 2 Department of paramedical studies, HAN University of Applied Sciences, Nijmegen, The Netherlands; 3 Department of Neurology and Clinical Neurophysiology, Donders Institute for Brain, Cognition and Behaviour, Radboud University Medical Center, Nijmegen, The Netherlands; 4 IQ Healthcare, Radboud Institute for Health Sciences, Radboud University Medical Center, Nijmegen, The Netherlands

**Correction to: BMC Musculoskelet Disord 2019 20:360**

**https://doi.org/10.1186/s12891-019-2741-7**

Following publication of the original article [1], the authors reported that the headers in Table 2 in their paper were omitted. The original article [1] has been updated.

The correct Table 2 is shown below.

**Table 2:** test-retest and interrater reproducibility of serratus anterior hand held dynamometer strenght testing

	*Test (N)*	*Retest (N)*	*Diff test-retest (N)*	*95% LoA (N)*	*ICC3.1*_*agreement*_	*SEM*_*agreement*_	*SDC*_*agreement*_
	*mean (SD)*	*mean (SD)*	*mean (SD)*		*(95% CI)*	*(N)*	*(N)*
*test-retest*							
Pos A	383.88 (77.65)	404.92 (84.96)	− 21.05 (60.22)	− 139.08; 96.98	0.712 (0.420; 0.871	44,10	122,40
Pos B	314.43 (75.1)	322.34 (82.34)	−7.90 (65.90)	− 137.06; 121.26	0.658 (0.324; 0.846)	45,80	127,00
Pos C	351,54 (100.36)	376.16 (93.55)	−24.62 (59.37)	− 140.99; 91.75	0.794 (0.490; 0.916)	44,52	123,40
	*Tester JI*	*Tester HK*	*Diff Tester JI* VS *HK*				
	*mean (SD)*	*mean (SD)*	*mean (SD)*				
*Interrater*							
Pos A	383.88 (77.65)	340.44 (82.95)	43.43* (92.74)	− 138.34; 225.20	0.794; (0.552; 0.912)	55,80	196,80
*Pos B*	314.43 (75.10)	264.21 (61.76)	50.21* (78.85)	− 104.34; 204.76	0.277 (−0.089; 0.605)	55,80	154,50
Pos C	351.54 (100.36)	265.10 (40,0))	86.48* (86.21)	−82.49; 255.45	0.226 (−0.107; 559)	85,31	236,47

Legend: *ICC 3.1* intraclass correlation coefficient model 3.1, *95%CI* 95% confidence interval, *LL* Lower limit, *UL* upper limit

*N* Newton, *SD* Standard deviation, *Diff* Difference, *LoA* limits of agreement, *ICC* Intraclass correlation coefficient, *CI* Confidence interval, *SEM* Standard error of measurement, *SDC* Smallest detectable change, *%* percentage

*: *p* < 0.001
